# Supplementary material for: 2D transition metal dichalcogenides with glucan multivalency for antibody-free pathogen recognition
Source: Nat Commun. 2018 Jun 29;9:2549. doi: 10.1038/s41467-018-04997-w (PMC6026184; doi:10.1038/s41467-018-04997-w)
Supplement: Supplementary file 1 — Supplementary Information [file 41467_2018_4997_MOESM1_ESM.pdf]

Supplementary Information

2D Transition Metal Dichalcogenides with Glucan Multivalency for Antibody-free Pathogen Recognition

by Kang T. W. et al.

Supplementary Methods

Materials

Transition metal dichalcogenides (TMDs) and polyethylene glycol MW 35,000 (PEG) were purchased from Alfa Aesar. Dextran MW 40,000 (denoted 'dex'), dextran MW 1000 (dex1000), and glucose were purchased from Sigma Aldrich. *Escherichia coli* O157:H7 (*E. coli*) and *Staphylococcus* (*S.*) *aureus* were received from Korean Agricultural Culture Collection (KACC), and *Salmonella* typhimurium was purchased from ATCC. *E. coli* K-12 and *E. coli* O1:K1:H7 were purchased from Korean Culture Center of Microorganisms (KCCM). *E. coli* O157:H7-specific antibody was purchased from Abnova (Catalog No. PAB 13942).

Instruments

AFM (XE-100, Park Systems), FT-IR spectrometer (IS10, Thermofisher), and UV-Vis spectrophotometer (UV-2600, Shimadzu) were used for characterization of dextran, dex-TMDs and PEG-TMDs. Photoluminescence and Raman of dex-TMDs were measured by using a Raman and PL microscope (UniRAM, Uninanotech), and the excitation/emission profiles of dex-TMDs were measured by a spectrofluorometer (NanoLog, HORIBA). The affinity of dex-WS_2 and an *E. coli* O157:H7-specific antibody against *E. coli* O157:H7 were measured by a quartz crystal microbalance (QCM) instrument (QSence-E4).

Characterization of dex-TMDs

For analysis of dex-TMDs with Raman and XPS, 10 μL of as-prepared dex-TMDs were dropped on a Si wafer and dried at room temperature for 4 h. Then, the Raman or XPS spectra of dex-TMDs were measured. The PL of dex-TMDs were measured under excitation at 532 nm (9 W). For FT-IR analysis, dex-TMDs were lyophilized for 24 h, and then the FT-IR spectra of dex-TMDs

(10 mg) were obtained using an ATR module. To obtain the TEM images of dex-TMDs, 100 μL of the dex-TMDs solution was diluted with 10 mL of water. A 10 μL of the resulting solution was dropped on a Cu grid, followed by drying for 12 h at room temperature.

Exfoliation of TMDs with PEG

A 6 g portion of TMD (8.4 g for WSe_2) was added into 200 mL of a PEG aqueous solution (2 g L^{-1}), which was then sonicated in an ice bath for 5 h by using a tip sonicator with a 75% amplitude and a pulse sequence of 6 sec-on and 2 sec-off. The resulting solution was centrifuged at 1977 xg for 1.5 h to discard unexfoliated TMD. Then, the supernatant was collected and re-centrifuged at 15,344 xg for 1.5 h to obtain the sediment. The collected sediment was re-dispersed in water (25 mL) and centrifuged at 3024 xg for 1.5 h. The supernatant (3024-15,355 xg) was obtained and used for further experiments.

Bacteria culture

Escherichia coli O157:H7 (*E. coli*), *Salmonella typhimurium* and *Staphylococcus* (*S.*) *aureus* were cultured with trypticase soy broth (TSB) solution for 24 h at 37ºC in a shaking incubator. After then, the bacteria were centrifuged at 1977 xg for 5 min at 4°C, and the obtained bacteria were suspended in phosphate buffered saline (PBS, 10 mM, pH 7.4). The concentration of the bacteria were counted on a C-chip using an optical microscope. The final concentration of bacteria was adjusted to approximately 1×10^8 CFU mL^{-1} before use.

Calculation of the affinity (K_d) of dex-WS_2 against *E. coli*

For the calculation of the affinity of dex-WS_2 to *E. coli*, the number of W atoms in a dex-WS_2 square nanosheet was calculated by considering their lateral size of 50 nm and lattice space of 0.27

nm. The distance between W-W was 0.311 nm, which was calculated from the lattice space. Each dex-WS_2 nanosheet has 21,600 W atoms.

The affinity of dex-WS_2 against *E. coli* was calculated by the Langmuir isotherm equation. A fractional coverage θ is shown below:

$$\theta = \frac{K \cdot p}{1 + K \cdot p} \tag{1}$$

K is an association constant where $K = \frac{k_a}{k_d}$, k_a is an adsorption constant and k_d is a desorption constant, and p is pressure. In the dex-WS_2 system, the fractional coverage corresponds to I/I_∞ where I is the Raman intensity of bound dex-WS_2 at a certain concentration and I_∞ is the Raman intensity of dex-WS_2 fully covering on the surface of the bacteria. In addition, the pressure is equivalent to the concentration of dex-WS_2 nanosheets, C, which gives the following equation:

$$\frac{I}{I_\infty} = \frac{K \cdot C}{1 + K \cdot C} \tag{2}$$

$$\frac{C}{I} = \frac{C}{I_\infty} + \frac{1}{K \cdot I_\infty} \tag{3}$$

The above equation was applied to the plot of the Raman intensity with the concentration of dex-WS_2. By obtaining the slope and the y intercept, we calculated the association constant (K). The reciprocal of K gave the dissociation constant (K_d) of dex-WS_2 to *E. coli*.

Affinity Measurement of dex-WS_2 and *E. coli* O157:H7-specific antibody using a QCM

The affinity of dex-WS_2 and an *E. coli* O157:H7-specific antibody against *E. coli* O157:H7 using a quartz crystal microbalance (QCM) instrument was measured. After self-assembling 11-

mercaptoundecanoic acid (MUA, 10 mM) and 11-hydroxy-1-undecanethiol (HUT, 50 mM) for 8 h at 40 ^{o}C on a gold QCM chip, the carboxyl group of MUA on the chip was activated by EDC/NHS (10 mM EDC and 20 mM NHS) for 1 h. Then, an antimicrobial peptide (KNYSSSISSIHC) (20 mL, 0.1 mg mL^{-1}) was added into the chip and reacted for 24 h to immobilize *E. coli* O157:H7 on the QCM chip. After washing the chip with DI water and EtOH, the bacteria solution (5 × 10^{6} CFU mL^{-1}) flowed into the chip at a rate of 50 μL min^{-1} for 30 min. After washing it with PBS for 30 min, dex-WS_2 or the antibody flowed into the QCM chip by changing its concentration from 2 to 16 nM for 30 min. Finally, the chip was washed with PBS for 30 min.

Detection of a single copy of bacteria with dex-WS_2

A 100 μL portion of dex-TMD (125 μg mL^{-1}) was dispersed in 500 μL of phosphate buffered saline (PBS, 10 mM, pH 7.4). Then, a 50 μL portion of bacteria was added into the dex-TMD solution in which the final concentration of bacteria was 10^{8} CFU mL^{-1}. The resulting mixture was incubated for 1 h at 25°C (500 rpm, Thermomixer). The reaction mixture was then centrifuged for 5 min (4°C, 1977 xg) to collect the bacteria as well as to remove unbound dex-TMDs. The collected bacteria was then washed with PBS several times to further remove unbound dex-TMD. After addition of 1 mL PBS to the bacteria sediment, a 2 μL portion of the bacteria solution was dropped on glass microarrays for measurement.

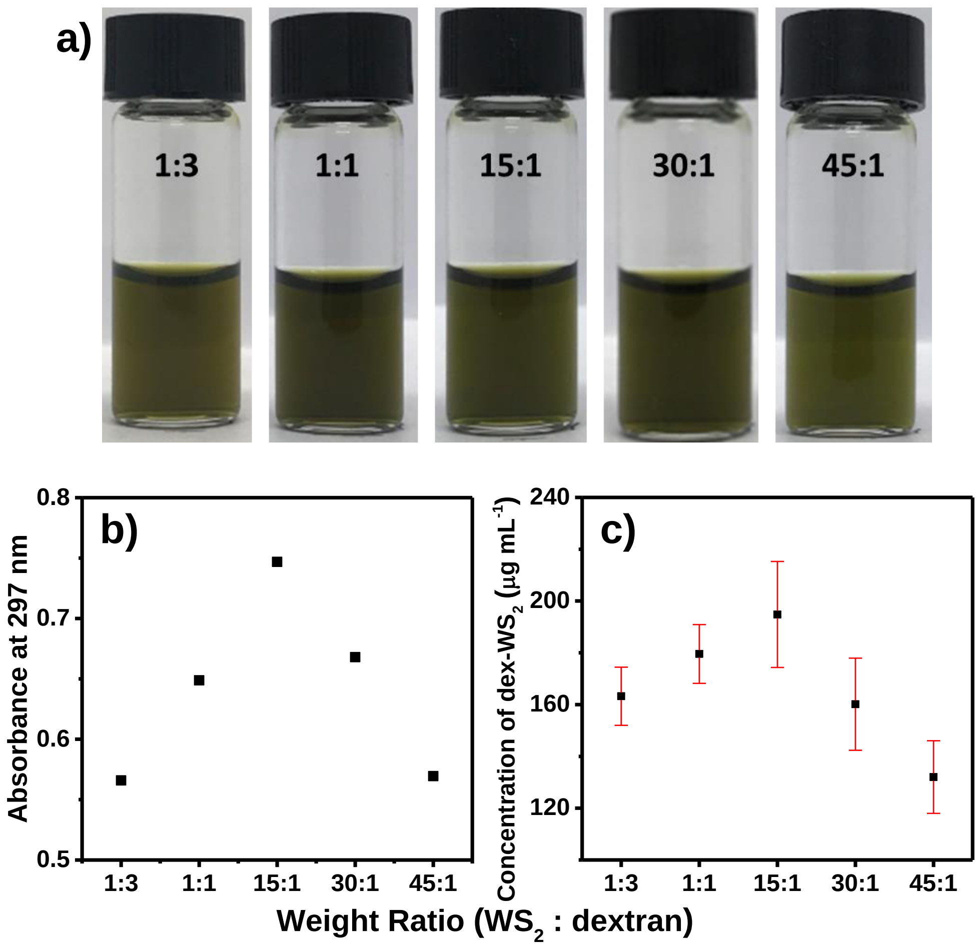

Supplementary Figure 1. Effect of the weight ratio of bulk WS_2 with dextran on the exfoliation efficiency of dex-WS_2. a) Optical photographs of the solution of dex-WS_2 nanosheets exfoliated at the various ratios of bulk WS_2 with dextran. b) Plot of the absorbance (297 nm) of the dex-WS_2 solutions with the ratios of bulk WS_2 with dextran. c) ICP-AES-measured concentrations of the dex-WS_2 nanosheets exfoliated at the different ratios of bulk WS_2 with dextran. All error bars represent a standard deviation from the mean values (n = 4).

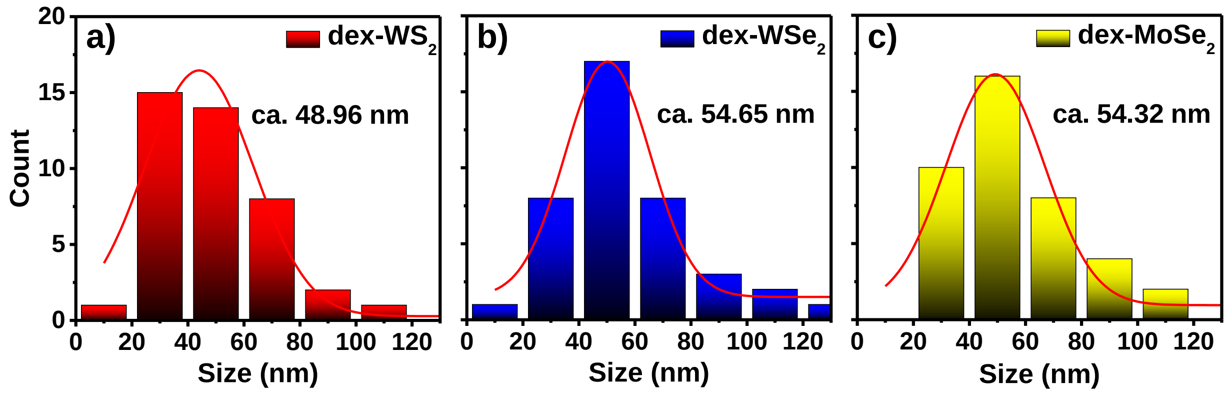

Supplementary Figure 2. Lateral size distribution of dex-TMDs measured by TEM. Size distribution of a) dex-WS_2, b) dex-WSe_2, and c) dex-$MoSe_2$.

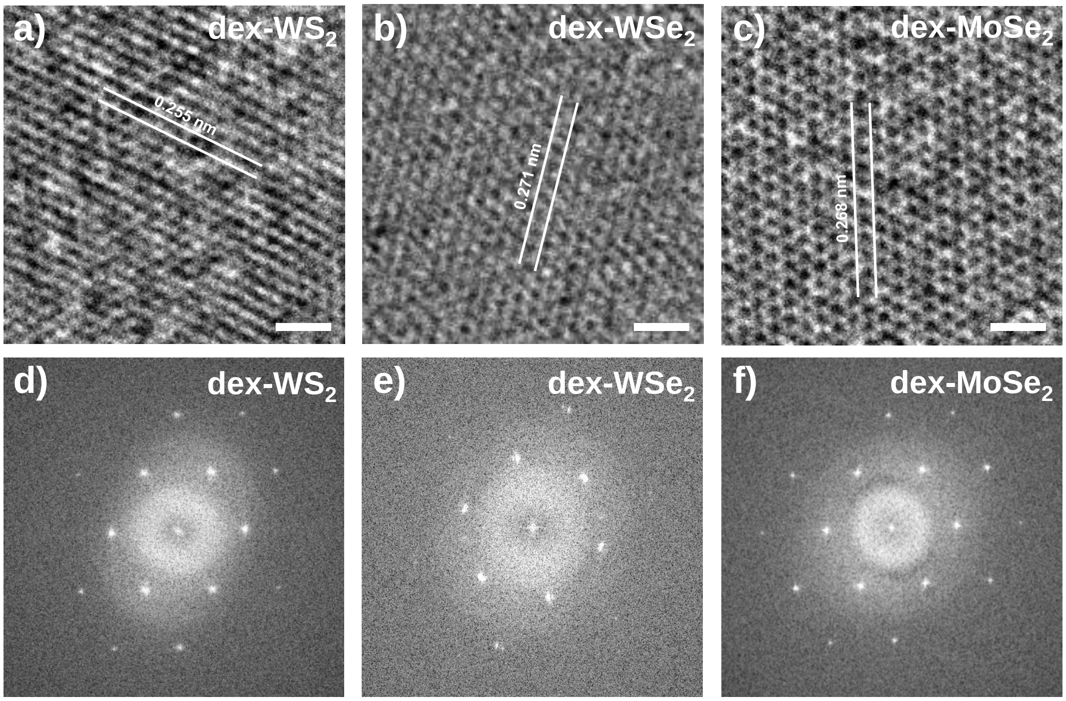

Supplementary Figure 3. HR-TEM images and SAED patterns of dex-TMDs. HR-TEM images of a) dex-WS_2, b) dex-WSe_2, and c) dex-$MoSe_2$ (scale bar, 1 nm). SAED patterns of d) dex-WS_2, e) dex-WSe_2, and f) dex-$MoSe_2$.

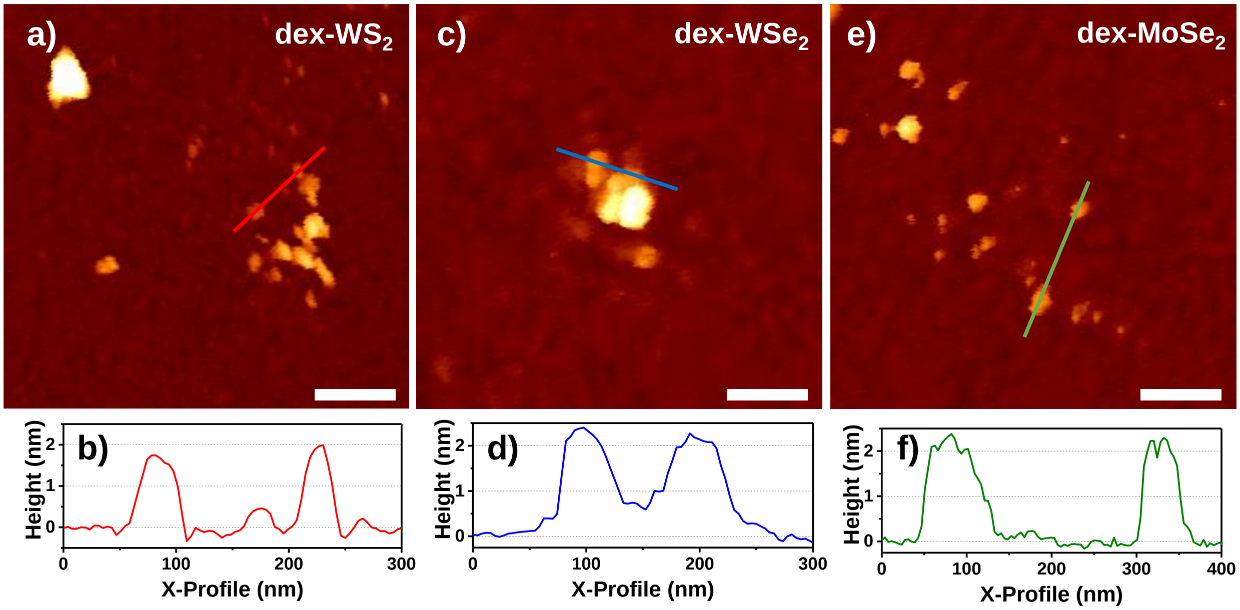

Supplementary Figure 4. AFM images and height profiles of dex-TMDs. AFM images of a) dex-WS_2, c) dex-WSe_2, and e) dex-$MoSe_2$ (scale bar, 200 nm). Height profile of b) dex-WS_2, d) dex-WSe_2, and f) dex-$MoSe_2$.

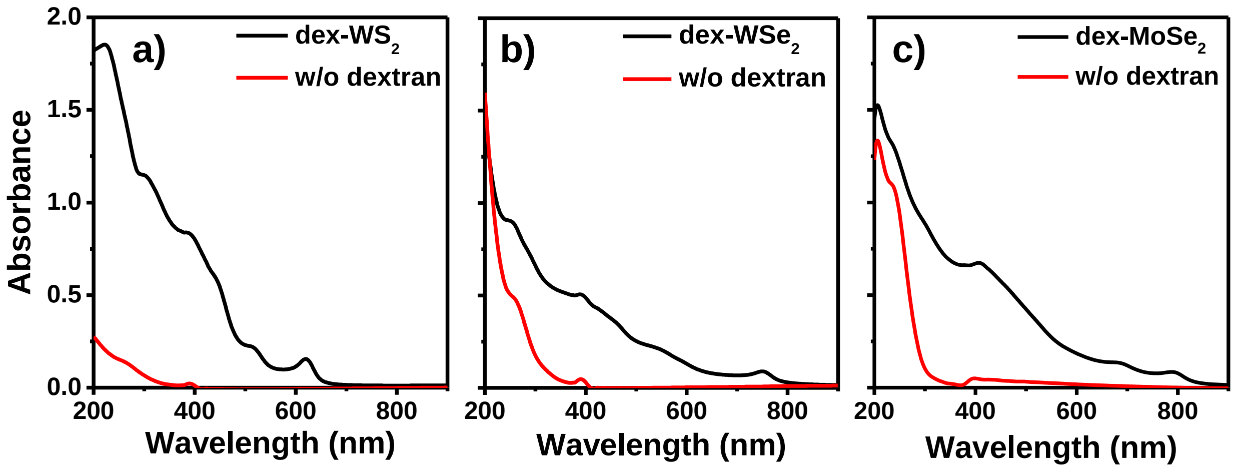

Supplementary Figure 5. UV-Vis extinction spectra of TMDs exfoliated with or without dextran. UV-Vis spectra of a) WS_2, b) WSe_2, and c) $MoSe_2$ with and without dextran.

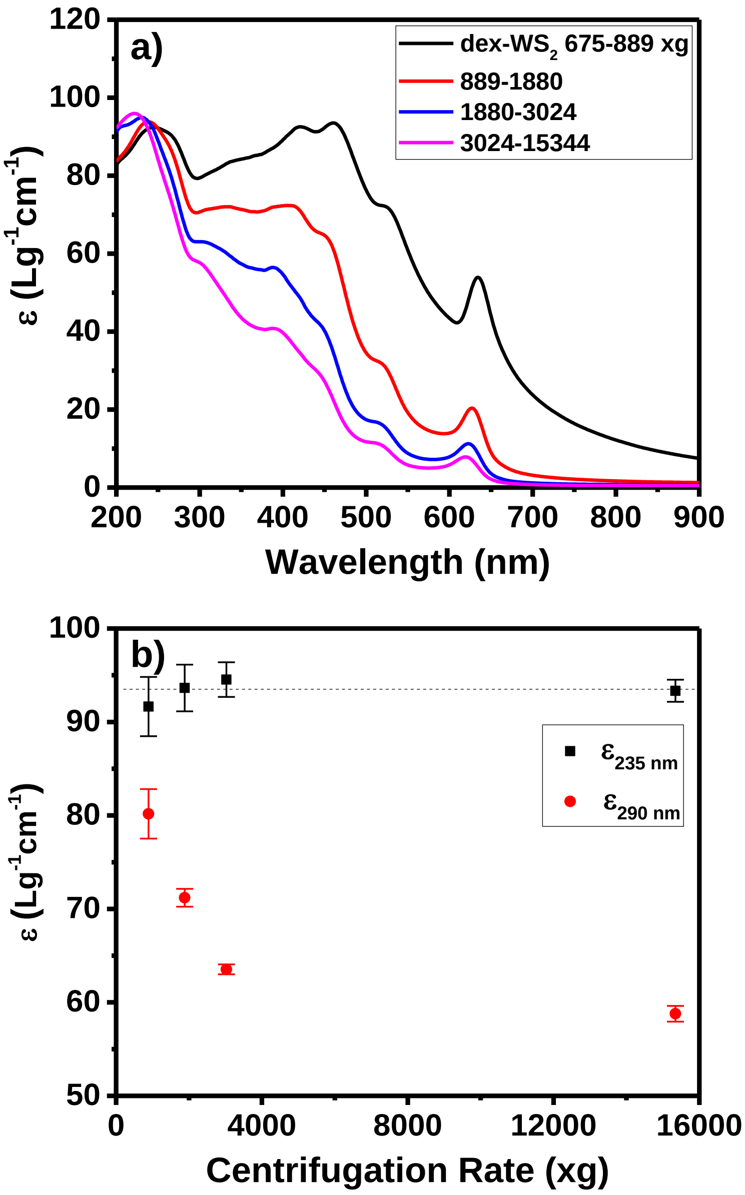

Supplementary Figure 6. Dependence of the extinction property of dex-WS_2 on centrifugation rates. a) Extinction spectra of dex-WS_2 obtained at various centrifugation rates. b) Plot of extinction coefficients at 235 and 290 nm against centrifugation rates. The mean value of the extinction coefficient at 235 nm is 93.45 L g^{-1} cm^{-1}. All error bars represent a standard deviation from the mean values (n = 4).

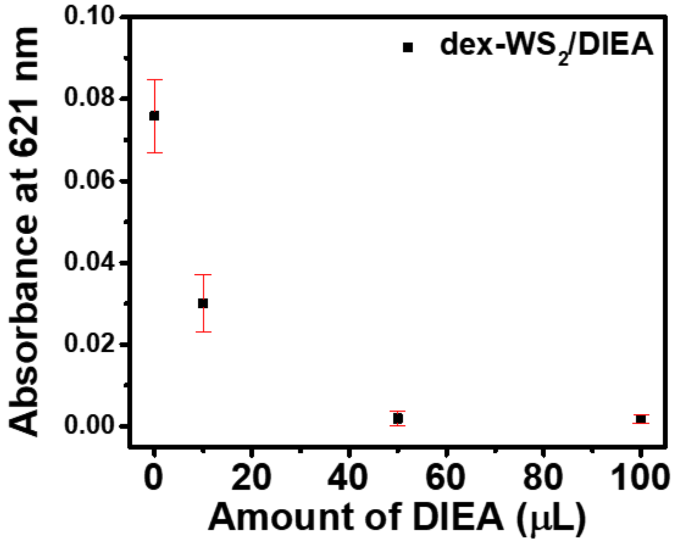

Supplementary Figure 7. Plot of the A excitonic absorption of dex-WS_2 at 621 nm with a DIEA concentration. All error bars represent a standard deviation from the mean values (n = 4).

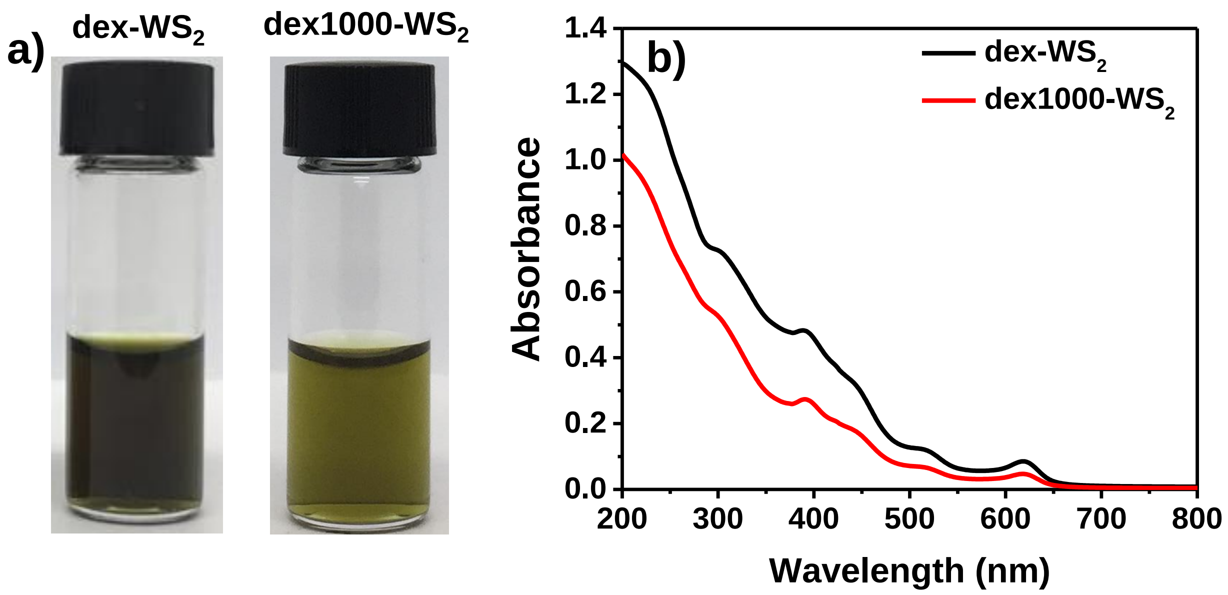

Supplementary Figure 8. Effect of the molecular weight of dextran on the efficiency of exfoliation in an aqueous solution. a) Optical photographs of the solution of WS_2 nanosheets exfoliated by dex and dex1000. b) UV-Vis extinction spectra of dex-WS_2 and dex1000-WS_2 nanosheets.

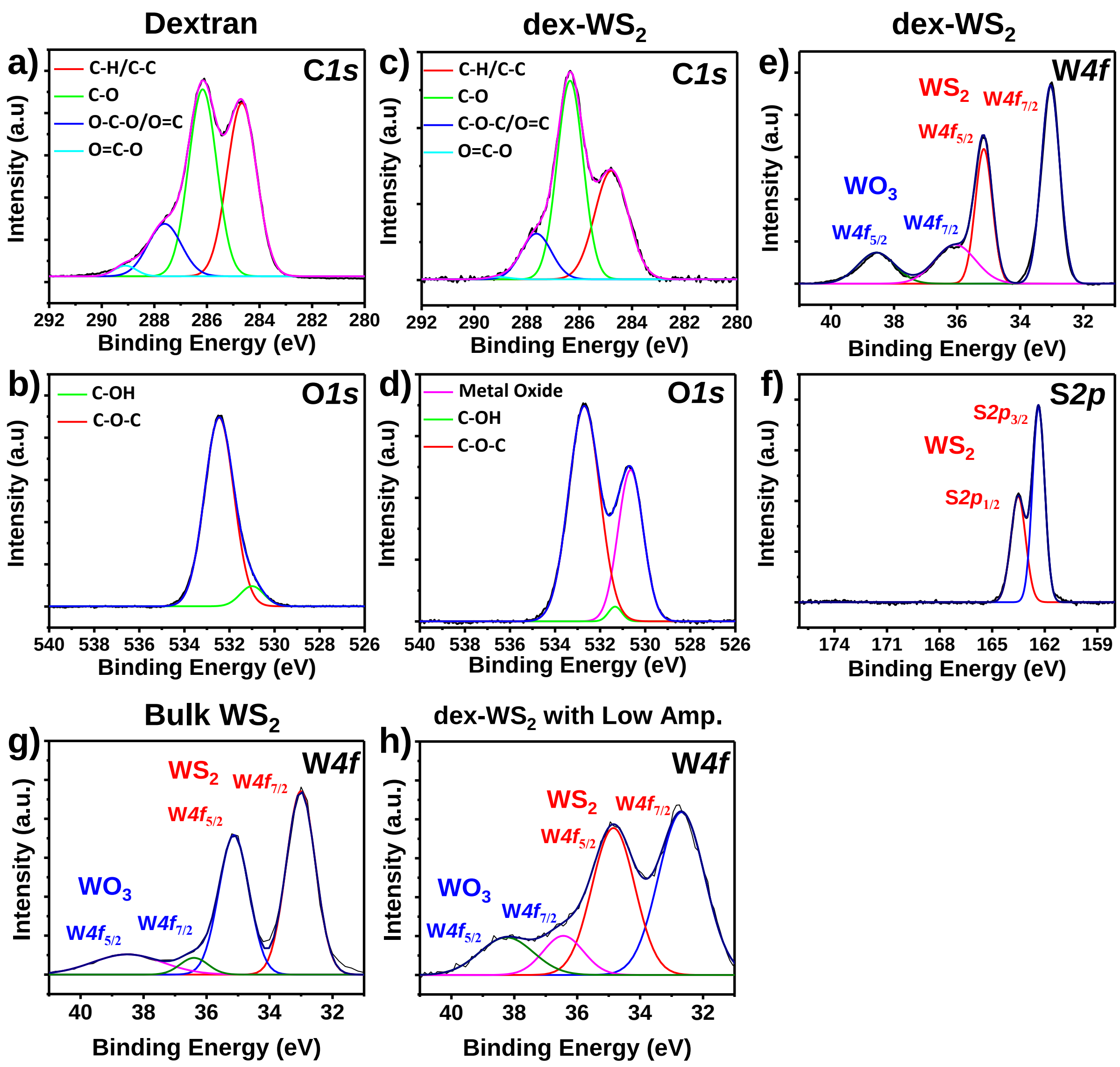

Supplementary Figure 9. XPS analysis of dextran, bulk WS_2, and dex-WS_2. a) C*1s*, and b) O*1s* spectra of dextran. c) C*1s*, d) O*1s*, e) W*4f*, and f) S*2p* spectra of dex-WS_2 nanosheets. g) W*4f* spectrum of bulk WS_2. h) W*4f* spectrum of dex-WS_2 exfoliated at a lower amplitude (37.5%).

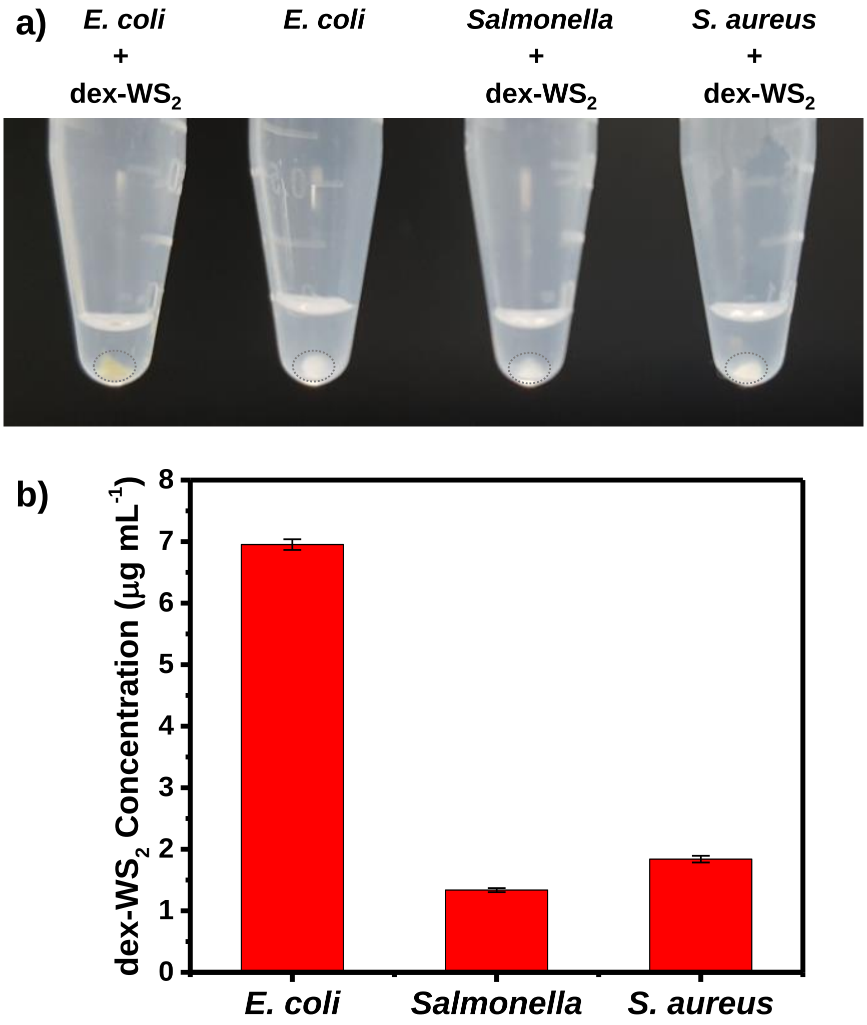

Supplementary Figure 10. Additional confirmation of the selective binding of dex-WS_2 on *E. coli* O157:H7. a) Optical photograph of a pile of bacteria before and after reaction with dex-WS_2. b) Concentration of WS_2 bound on the dex-WS_2-treated bacteria using ICP-AES. All error bars represent a standard deviation from the mean values (n = 4).

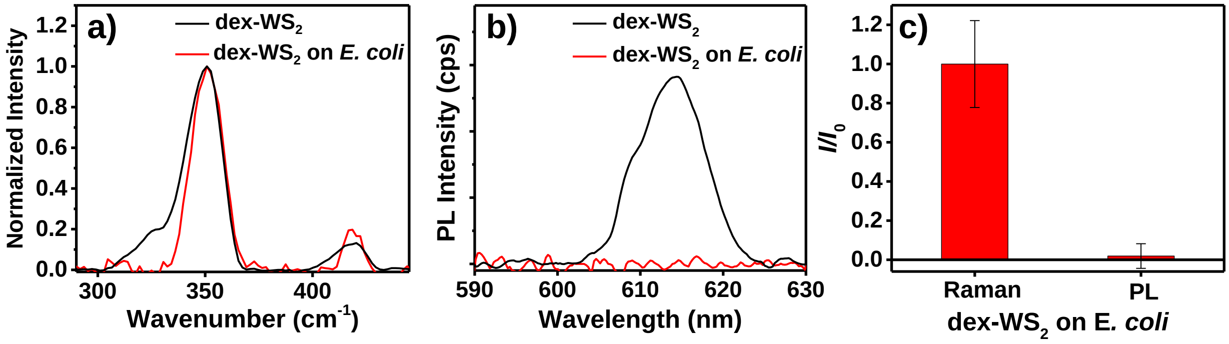

Supplementary Figure 11. Optical properties of dex-WS_2 before and after binding on *E. coli*. a) Raman and b) PL spectra of dex-WS_2 before and after binding with *E. coli*. c) Intensity ratio of the Raman and PL spectra of dex-WS_2 after and before binding onto *E. coli*. I_0 is the intensity of dex-WS_2 before binding, and I is its intensity after binding. All error bars represent a standard deviation from the mean values (n = 4).

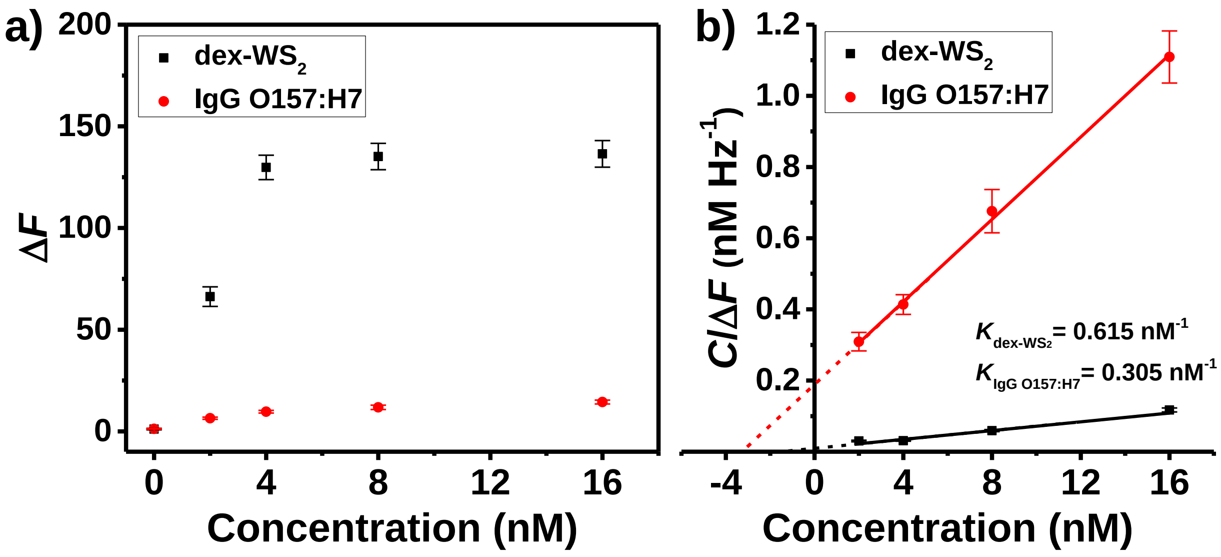

Supplementary Figure 12. Measurement of the binding affinity of dex-WS_2 and an *E. coli* O157:H7-specific antibody against *E. coli* O157:H7 using a quartz crystal microbalance (QCM). a) Frequency change as a function of the concentration of dex-WS_2 or an *E. coli* O157:H7-specific antibody. b) Langmuir isotherm for the binding of dex-WS_2 or an *E. coli* O157:H7-specific antibody to *E. coli* O157:H7. All error bars represent a standard deviation from the mean values (n = 4).

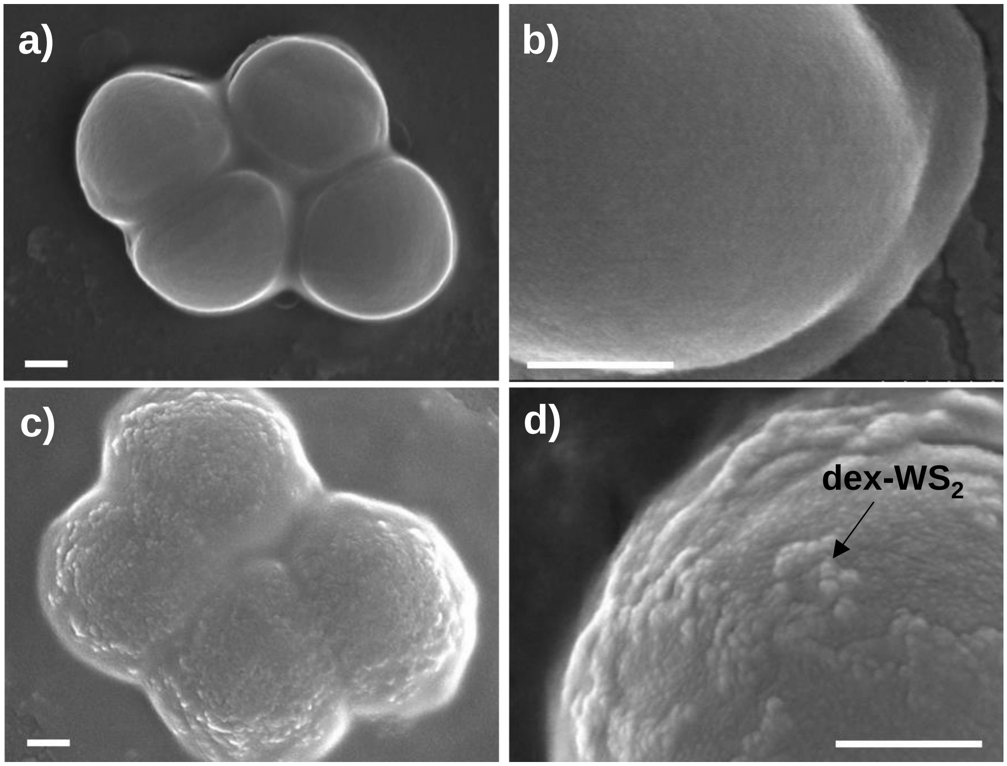

Supplementary Figure 13. SEM images of *E. coli* O157:H7 before and after reaction with dex-WS_2. a)-b) before treatment with dex-WS_2, and c)-d) after treatment with dex-WS_2 (scale bar, 200 nm).
